# Asymptomatic tuberculosis among contacts of people with tuberculosis in a programmatic study in Brazil

**DOI:** 10.36416/1806-3756/e20260306

**Published:** 2026-11-09

**Authors:** Guilherme Senos Tavares dos Santos, Dinah Carvalho Cordeiro, Aline Andries e Arantes, Elisa Barroso de Aguiar, Daniel Souza Sacramento, Bernardo Moutinho Gomes, Cícero Rogerio da Silva Matos, Pedro Kuabara, Lucas Maia Portugal, Anete Trajman

**Affiliations:** 1. Universidade Federal do Rio de Janeiro - UFRJ - Rio de Janeiro(RJ), Brasil.; 2. Secretaria Municipal de Saúde, Manaus (AM), Brasil.; 3. McGill TB International Centre, McGill University, Montreal (QC), Canada.

## TO THE EDITOR:

More than 2.4 million cases of tuberculosis are undiagnosed each year, raising concern about tuberculosis elimination.^1^ Undiagnosed tuberculosis may result in worse outcomes in the short and long terms, as well as in sustained transmission. Tuberculosis is currently defined as the presence of tissue injury by viable *Mycobacterium tuberculosis* with or without symptoms and with or without bacteriological confirmation.^2^ Asymptomatic tuberculosis poses a special problem for early diagnosis. Historically, tuberculosis screening has been strongly linked to the identification of symptomatic individuals. However, in an individual patient data meta-analysis of 12 population-based surveys from 12 countries, 62.5% of 1,944 individual**s** with tuberculosis in community settings did not report cough, and up to 27.7% had no symptoms at all.^3^ Thus, screening restricted to persons with symptoms may be ineffective. 

The prevalence of asymptomatic tuberculosis varies according to the setting and the population being evaluated. In a meta-analysis conducted before the literature debate on subclinical/asymptomatic disease, it was suggested that the prevalence of tuberculosis among contacts in high-burden countries was 3.1%.^4^ However, little is known about the prevalence of asymptomatic disease in this population. 

The ExpandTPT project^5^ was aimed at scaling up tuberculosis preventive treatment among household and close contacts of people with tuberculosis in eight high-burden cities in Brazil. Among interventions, health care workers and community health workers were trained to identify contacts and refer them to a health facility to be screened for the four cardinal symptoms (cough, fever, night sweats, and weight loss) while the workers themselves were also screened for tuberculosis infection. 

For the ExpandTPT project, we analyzed the proportion (and 95% confidence intervals) of contacts with tuberculosis and, among them, those with and without symptoms. The project was carried out at 327 primary care clinics in five cities (phase 1) between April of 2023 and June of 2024, and at 54 primary care clinics in three cities (phase 2) between July of 2024 and October of 2025. Of those eight cities, the cities of São Paulo, in the state of São Paulo, Rio de Janeiro, in the state of Rio de Janeiro, Manaus, in the state of Amazonas, and Recife, in the state of Pernambuco, from phase 1 and the city of João Pessoa, in the state of Paraíba, from phase 2 sent data for analysis. We extracted data from the contact registry books implemented as part of the project, with data being routinely collected by the staff of the clinics, including information on symptoms. No standardized symptom questionnaire or form was applied; data reflected how information on symptoms was collected in pragmatic conditions.

Only those clinics that were willing to share the anonymized data were included. The presence of any symptom was collected as a single categorical variable (yes or no). Health care workers were instructed to fill in *yes* if any of the four cardinal symptoms were present. A diagnosis of pulmonary tuberculosis was accepted if contacts were prescribed antituberculosis drugs; that is, either a bacteriologically confirmed diagnosis (by any method) or a clinical diagnosis (with or without radiological signs of tuberculosis) was accepted. The study was approved by the local institutional review board (Protocol no. 67116923.3.0000.5257). 

We gathered data on 1,529 identified contacts of 1,056 index persons with pulmonary tuberculosis at 112 clinics. Of the 1,529 contacts that were evaluated, 93 (6.1%; 95% CI, 4.9-7.3%) were diagnosed with tuberculosis (with 55 being diagnosed clinically and 38 being diagnosed with bacteriological confirmation) and were prescribed antituberculosis regimens. Of those, one had missing data on symptoms and 40/92 (43.5%; 95% CI, 33.3-53.6%) were reported as being asymptomatic by the health care workers. 

Of the 127 contacts who reported symptoms, 52 (40.9%; 95% CI, 32.3-49.9%) were diagnosed with tuberculosis, whereas 40 (3.3%; 95% CI, 2.4-4.4%) of the 1,211 asymptomatic contacts were diagnosed with tuberculosis. A clinical diagnosis was more common among asymptomatic contacts (50%; 95% CI, 34.5-65.5%). 

We concluded that tuberculosis was highly prevalent among contacts presenting to primary care clinics (i.e., a prevalence of 6.1%). More importantly, a high proportion of contacts with a final diagnosis of tuberculosis (i.e., 43.5%) did not report symptoms in the primary care setting, as per routine evaluation. 

The present study has limitations. Data sharing was not mandatory, and this can result in selection bias. In addition, a high proportion of tuberculosis diagnoses, especially in persons without symptoms, were not bacteriologically confirmed, raising the possibility of classification bias. Symptom definition was based on pragmatic data collection, and symptoms may have been overlooked, thus resulting in classification bias (differential overdiagnosis in asymptomatic tuberculosis). On the other hand, the programmatic classification of symptoms may be a strength, given that it reflects the real-world scenario. Our findings suggest that screening for tuberculosis should not be restricted to contacts with symptoms and that health care workers may need enhanced training to capture symptoms and pursue a tuberculosis diagnosis, especially among contacts of persons with pulmonary tuberculosis. 
